# Effect of pelvic floor muscle training using mobile health applications for stress urinary incontinence in women: a systematic review

**DOI:** 10.1186/s12905-022-01985-7

**Published:** 2022-10-03

**Authors:** Yuqing Hou, Suwen Feng, Baoqin Tong, Shuping Lu, Ying Jin

**Affiliations:** Gynecology Department, Women’s Hospital School of Medicine Zhejiang University, No. 1 Xueshi Road, Hangzhou, 310006 Zhejiang Province China

**Keywords:** MHealth app, Pelvic floor, PFMT, SUI, MUI

## Abstract

**Background:**

Pelvic floor muscle training (PFMT) is the first-line treatment for urinary incontinence, but adherence to PFMT is generally poor. Currently, a number of novel strategies exist to facilitate adherence of PFMT. We sought to determine effectiveness of mHealth app-based PFMT for treating stress urinary incontinence (SUI) or stress-predominant mixed urinary incontinence (MUI) in women. The primary objective was to assess the effects of mHealth app-based PFMT and usual treatment on severity of the symptom, the quality of life (QoL) of users and the patient’s global impression of improvement. The secondary objective was to assess how mHealth app use affects adherence of PFMT.

**Methods:**

All randomized controlled trials and quasi-randomized controlled trials aimed at evaluating the effects of mHealth app-based PFMT in women with SUI or stress-predominant MUI were included. Twelve electronic databases, namely the Cochrane Library, PubMed, CINAHL, Embase, Web of science, OVID, SciELO, REHABDATA, PEDro database, Chinese CNKI and Wanfang and the Open Grey databases were used as search sources. The protocol was registered in PROSPERO (CRD 42020183515). This systematic review was developed following the PRISMA 2020 Checklist. The Cochrane Handbook for Systematic Reviews of Interventions for Randomized Controlled Trials was used to assess risk of bias in included studies. Two authors extracted the data into a standardized spreadsheet.

**Results:**

Six studies that met the eligibility criteria were included. The full sample included 439 patients with treatment via mHealth app and 442 controls of usual treatment. ICIQ-UI SF, ICIQ-VS, and QUID scores decreased after follow-up in the mHealth app and control groups in six studies. ICIQ-LUTS QoL scores decreased after follow-up in the mHealth app and control groups in three studies. In only one study, ICIQ-VS QoL scores decreased after 1 month and 2 months of follow-up in the mHealth app group, but increased abruptly after 3 months of follow-up. EQ5D-VAS scores increased in both groups in one study. The percentage of PGI-I increased in the mHealth app group in three studies after follow-up. After follow-up in three studies, BPMSES scores and self-reported adherence scores increased in the mHealth app group relative to the initial time point, but in one study, at 6 months compared with 3 months of follow-up, adherence scores decreased slightly in the mHealth app group.

**Conclusions:**

This systematic review determined that mHealth app-based PFMT showed promise from the perspective of improving both outcomes and exercise adherence.

## Introduction

SUI is defined by the International Continence Society as “complaint of involuntary loss of urine on effort or physical exertion (e.g., sporting activities), or on sneezing or coughing” [1], and it is common among women and often leads to a significant decline in their QoL [2]. Accumulating epidemiological evidence indicated that the prevalence of SUI across studies varied from 10 to 39% and increased with age [3–5]. Data released by the United States Census Bureau recently showed that the demand for care for pelvic floor disorders will increase by 35% between 2010 and 2030 [6].

Evidence-based incontinence treatment can be separated broadly into stress incontinence surgery, medications, behavior and lifestyle modification, with the PFMT most commonly undertaken [7]. PFMT is the first-line conservative management programme for SUI with no adverse reaction [8]. The ideal treatment requires patients to be instructed to perform the exercises properly and persistently commit to it [9–11]. There is evidence that women perform better with exercise regimes supervised by medical staff and supervised PFMT showed satisfactory results in alleviating SUI symptoms [12, 13]. However, supervised PFMT is time-consuming, costly, and requires frequent visits to relevant medical institutions, which may hinder long-term treatment adherence. Adherence is considered crucial to PFMT success [14]. Unfortunately, treatment adherence to PFMT is still poor [15].

Mobile health applications (mHealth app) are increasingly being used in health care and public health practice for patient communication, monitoring, education, and to promote adherence to chronic diseases management [16]. A cross-sectional study of 200 adult women in Pennsylvania showed high rates of overall mHealth app ownership, with smartphones accounting for 92%, and women with pelvic floor disorders have high capability of using mHealth app to communicate with their health care providers [17]. MHealth app for PFMT are personal care apps that assist users in training their pelvic floor muscles. A recent systematic review including three eligible studies showed that mHealth app-based PFMT can reduce urinary symptoms [18].

Therefore, this systematic review extends the current literature providing meta-analysis of the most recent RCTs and quasi-randomized controlled trials evaluating mHealth app to deliver PFMT for SUI or stress-predominant MUI. The primary objective was to assess the effects of mHealth app-based PFMT and usual treatment on symptom severity, the QoL of users and the patient’s global impression of improvement. The secondary objective was to assess how mHealth app use affects adherence of PFMT.

## Materials and methods

### Protocol and registration

This systematic review was developed following the PRISMA 2020 Checklist [19]. The systematic review protocol was registered in the PROSPERO database under number CRD 42020183515.

### Study design and eligibility criteria

This systematic review aimed to answer the following guiding question based on the PICO strategy: “Do women with SUI or stress-predominant MUI (P) who use mHealth app for PFMT (I) have better results (O) when compared with women using the usual treatment (C)?”. (1) Participants: participants were women who were diagnosed with SUI or stress-predominant MUI; (2) Intervention: in the intervention group, participants received mHealth app-based PFMT to help women treat or prevent SUI or stress-predominant MUI; (3) Comparison: traditional care (e.g., conventional home-based training without mHealth app) or no treatment in the control group; (4) Outcome: one or more of the following interesting outcomes have been reported (e.g., the severity of symptoms, QoL and the patient’s global impression of improvement were included as main outcome indicators; a secondary outcome measure was adherence to PFMT).

All RCTs and quasi-randomized controlled trials were included and there were no restrictions on year, language, publication status and type of setting.

The studies about qualitative studies, observational studies, review studies, case reports, case control studies, cohort studies, letters to the editor, conference abstracts, personal opinions, and books or book chapters were excluded.

### Sources of information and search

We searched the Cochrane Library, PubMed, CINAHL, Embase, Web of science, OVID, SciELO, REHABDATA, PEDro database, Chinese CNKI, Chinese Wanfang, and the Open Grey databases, a total of twelve electronic databases, each from their date of inception to October 2021. Our search strategy involved a combination of the following MeSH and free word [PubMed for example, (“Urinary Incontinence” OR “Stress Urinary Incontinence”) AND (“Pelvic Floor” OR “Pelvic Floor Muscle” OR “Pelvic Floor Muscle Training”) AND (“Woman” OR “Women” OR “Girl” OR “Female”) AND (“Mobile” OR “Portable” OR “Electronic” OR “eHealth” OR “mHealth” OR “App” OR “Software” OR “Reminder Therapy” OR “Programme” OR “Program” OR “System” OR “phone” OR “smartphone” OR “application” OR “web-based”). A reviewer first drafted the search strategy and then defined it through discussions with team members. Two reviewers conducted an independent literature search and thoroughly checked the reference lists of included studies to avoid omitting relevant studies.

### Study selection

During the literature screening process, search results from different electronic databases were imported into EndNote Version X9. These studies were selected at three distinct stages. In the first stage, two reviewers performed a methodical analysis of all study titles independently, and titles that did not meet the eligibility criteria were removed.

In the second stage, two reviewers read the abstracts independently for the initial application of the eligibility criteria. Studies containing titles that met the study objectives but did not have abstracts and full text available were removed.

In the last stage, the preliminary eligible studies were assessed in full text to verify whether they met the eligibility criteria. When reviewers disagreed about a particular study, a third reviewer was consulted to make a final decision. All stages were performed to reduce the literature search bias and literature screening bias.

### Process of data collection and extraction

After the selection, we used structured forms to extract data from each study, such as authors, year, place of the study, sample characteristics including number of participants and age, SUI diagnosis, name of mHealth app used, control group, information contained in the mHealth app, timing of outcome measurement, reminder frequency and outcome measurement tools.

In the PROSPERO database, the systematic review protocol took adherence as the main outcome measure and incontinence severity as an additional measure. But when we read the relevant literature, we found that mHealth app-based PFMT should first be able to improve the severity of SUI symptoms, and then improve the patient's adherence on the basis of SUI symptom improvement, so that the research will make sense. If the study used adherence as the primary outcome measure, we do not know whether the SUI symptom severity improved if adherence improved. Therefore, after the formal start of the study, we included symptom severity as the primary outcome measure and adherence as the secondary outcome measure.

To assess the impact of mHealth app-based PFMT on SUI symptoms (primary outcomes), the following data were extracted: the assessment of SUI symptoms based on the ICIQ-UI SF, ICIQ-VS and QUID. The ICIQ-UI SF is developed for assessing the prevalence, severity and impact on quality of life. It includes three scored items and one non-scored item, the sum-scores for the ICIQ-UI SF with (total score 0–21). Higher scores indicate more severe symptoms [20]. The ICIQ-VS is a questionnaire for assessing a range of pelvic floor dysfunction symptoms such as bowel, vaginal, and sexual matters. It consists of 14 questions, including 3 separate domains: the vaginal symptom (score from 0 to 53), sexual question (score from 0 to 58) and quality of life (score from 0 to 10). The higher the scores are, the worse the severity of the symptoms is [21]. The QUID consists of two subscales to identify SUI and/or urge urinary incontinence. Each subscale consists of three items to measure the symptom severity of the respective type of UI. Each item includes 6 frequency-based response options, ranging from “none of the time” to “all of the time,” which are scored from 0 to 5 points. The higher the scores are, the worse the severity of the symptoms is [22]. To assess how mHealth app use affects the QoL of users (primary outcomes), the following data were extracted: assessment of QoL specific to the condition based on the ICIQ-LUTS QoL and ICIQ-VS QoL. The ICIQ-LUTS QoL is a condition-specific quality of life questionnaire consisting of 19 items covering different aspects of everyday life that may be affected by leakage or other bladder conditions. These scores add up to a total of 19 to 76 points. A higher score indicates worser QoL [23]. The ICIQ-VS QoL is a questionnaire for assessing a range of pelvic floor dysfunction symptoms such as bowel, vaginal and sexual matters. It is composed of 14 questions, divided into 3 independent domains: the domain of the vaginal symptom, sexual question, and quality of life. The higher the scores are, the worse the severity of the symptoms is [21]. And the assessment of health specific QoL based on the EQ5D-VAS, a vertical VAS with the endpoints 0 (worst imaginable health state) and 100 (best imaginable health state). The higher scores indicate better QoL [24]. To assess the impact of mHealth app use on the patient's global impression of improvement (primary outcomes) through the PGI-I. PGI-I is a validated questionnaire asking the participants to rate their current condition compared to pre-treatment status. There are seven response options, including very much better, much better, a little better, no change, a little worse, much worse, very much worse [25].

Assessing how mHealth app use affects adherence to PFMT (secondary outcome), the following data were extracted: the assessment of the adherence based on the BPMSES and self-reported adherence (from 0 to 10, regarding their commitment to exercises where 0 means “no exercise at all” and 10 means “maximal adherence”).

We resolved any differences by discussions and when both reviewers disagreed, a third one was consulted to make a final decision. Where trial data were possibly collected but not reported, we sought further clarification from the trialists. We processed all included trial data as described in the Cochrane Handbook for Systematic Reviews of Interventions.

### Risk of individual bias of the studies

The “Cochrane Handbook for Systematic Reviews of Interventions Version 5.1.0” [26] was used to assess risk of bias for selected studies. Two authors assessed independently each domain regarding the potential risk of bias.

We considered random sequence generation, allocation concealment, blinding of participants and intervention implementers, blinding of outcome evaluator, incomplete outcome data, reporting bias and other bias and deemed each category at low, high or unclear risk of bias. Where there was insufficient information to make a clear decision, trials were rated at “unclear risk of bias”. If the research fully meets these criteria, the possibility of various biases is low, and the quality level is A grade; If the research partially meets these standards, the probability of bias is moderate, and the quality level is B grade; If these criteria are not met at all, the possibility of bias is high, and the quality level is C grade. Any disagreements were resolved by discussion.

### Summary measures and syntheses of results

The mean scores of the ICIQ-UI SF, ICIQ-VS, and QUID were described to assess the improvement of SUI symptoms. The impact of SUI on the QoL of the individuals was described from the mean scores of the ICIQ-LUTS QoL, ICIQ-VS QoL and EQ5D-VAS. The improvement based on PGI-I results were described by frequency (percentage). The impact of mHealth app-based PFMT on the adherence was described from the mean scores of the BPMSES and the self-reported adherence.

The mean scores of symptoms severity, QoL and adherence were compared between studies by calculating the standardized mean difference (SMD) using the method of Yange and Dalton [18]. The standardized difference was obtained by subtracting the mean post-intervention scores from the mean scores in the initial period of the study, which was weighted by the standard deviation of the between-group differences. Since the post-intervention period varied among eligible studies, the SMD was also weighted according to the number of months between the pre-intervention and post-intervention periods.

## Results

### Study selection

**Fig. 1:**
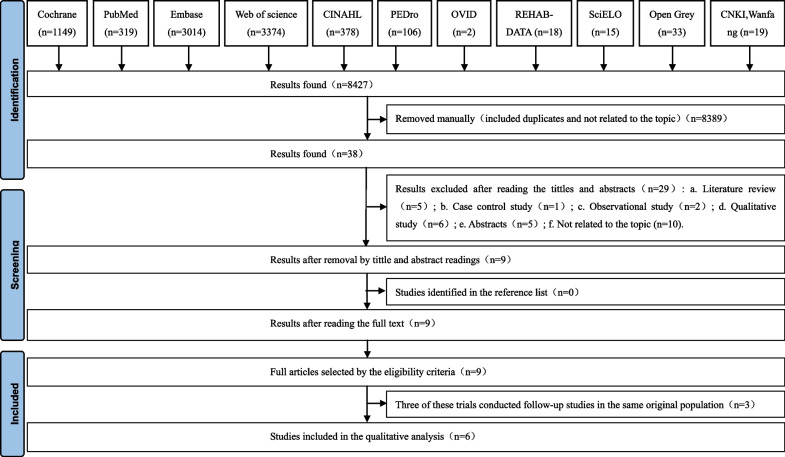
Flow diagram of the study selection process

We found five studies in English and one study in Chinese, all six studies in women with SUI. We ultimately included six studies that have important implications for current clinical practice and constructed summary tables of that evidence for SUI. Figure 1 illustrated the detailed process of search, identification, inclusion, and exclusion of studies.

### Characteristics of eligible studies

The summary of the main features of the studies can be found in Table 1. The studies were published between 2013 and 2021 and conducted in Sweden [27–30], Brazil [31], China [32, 33] and Netherlands [34, 35]. The full sample included 439 patients with treatment via mHealth app and 442 controls of usual treatment. Age ranged from 18 to 86 years and all studies informed the ethical criteria involved, including the use of a consent agreement. One study used the URinControl [34, 35], one study used the Tät.nu [27, 28], while another study used the Tät [29, 30]. And one study used the Diário Saúde [31], one study used the Hospital-Community-Family home care [32] and lastly, one study used the Pen Yi Kang [33]. All studies reported that mHealth app provided information about SUI and instructions about PFMT. The follow-up periods consisted in 1 month [31], 6 weeks [33], 2 months [31], 3 months [29, 31, 33], 4 months [27, 34], 6 months [32, 33], 1 year [28, 35] and 2 years [28, 30]. The reminders were sent three times per day [27–30, 32], twice per day [31], reminders frequency set by the participant [34, 35] and regularly audio reminders during the training [33].

**Table 1:** Summary of the main features and results of the eligible studies

Studies	Country	Sample	SUI diagnosis	Age, mean (SD)	App name	Comparative	App function	Timing of outcome measurement	Reminder frequency	Outcome measurement tools
Loohuis et al. [34, 35]	Netherlands	262 App: 131 Control: 131	≥ 2 UI episode/week	20–86 App: 53.2 ± 12.8 Control: 51.3 ± 10.3	URinControl	Participants in the usual care group were referred to their own general practitioner to discuss treatment options	Information on UI Instructions of PFMT; Reminder about PFMT Data analysis and feedback	At 4 months, 12 months	Reminder frequency set by yourself	ICIQ-UI SF ICIQ-LUTS QoL PGI-I
Wang et al. [33]	China	108 App: 54 Control: 54	≥ 1 SUI episode/month	23–34 App: 29.2 ± 2 Control: 9.1 ± 2.9	Pen Yi Kang	A 45-min pelvic floor rehabilitation education (through lectures, discussions, answer to queries.) and one-to-one PFMT practice guidance at baseline and before discharge	Instructions of PFMT; Reminder about PFMT	At 6 weeks, 3 months and 6 months postpartum	Regularly audio reminders during the training	ICIQ-UI SF BPMSES
Araujo et al. [31]	Brazil	33 App: 17 Control: 16	Women with self-reported SUI symptoms were included	App: 47.2 ± 10.6 Control: 53.3 ± 13.2	Diário Saúde	Printed instructions. The static image of muscular contraction presented in the paper was similar to that obtained through a sEMG screen	Visual component of sEMG as a guide for PFMT; An alarm that reminds the used;	At 1 month, 2 months and 3 months	Two reminders/day	ICIQ‐VS QUID ICIQ‐VS QoL Self-reported adherence
Jia et al. [32]	China	108 App: 51 Control: 54	Women with urinary incontinence investigated by ICIQ-SF and diagnosed as SUI through medical history and related examinations	App: 52.68 ± 13.07 Control: 51.68 ± 14.85	Hospital-Community-Family home care	Receive regular health education in incontinence care clinics, give verbal guidance on the causes, treatment methods, pelvic floor muscle exercise methods, frequency, precautions and importance of SUI, and issue health education prescriptions for PFMT	Information on SUI; Instructions of PFMT; Statistics about your training; Reminder about PFMT	At 6 months	Three reminders/day	ICIQ-UI SF BPMSES
Asklund et al. [29]	Sweden	123 App: 62 Control:61	≥ 1 SUI episode/week	27–72 App: 44.8 ± 9.7 Control: 44.7 ± 9.1	Tät	Postponed treatment and did not receive the app or any material included in the app during the study period	Information on SUI and pelvic floor; lifestyle; Instructions of PFMT; Statistics about your training; Reminder about PFMT	At the 3 months and 2 years	Three reminders/day	ICIQ-UI SF ICIQ-LUTS QoL PGI-I
Sjöström et al. [27]	Sweden	250 App: 124 Control: 126	≥ 1 SUI episode/week	18–70 App: 47.9 ± 10.6 Control: 49.4 ± 9.8	Tät.nu	Postal treatment	Information about SUI; and associated lifestyle factors. Instructions of PFMT; Statistics about your training Urotherapist support	At the 4 months, 1 year and 2 years	Three reminders/day	ICIQ-UI SF ICIQ-LUTS QoL PGI-I EQ5D-VAS

ICIQ‐UI SF, International Consultation on Incontinence Questionnaire Urinary Incontinence Short Form; ICIQ‐ LUTS QoL, ICIQ Lower Urinary Tract Symptoms Quality of Life; ICIQ‐VS, International Consultation on Incontinence Questionnaire—Vaginal Symptoms; PGI–I, Patient's Global Impression of Improvement; QUID, Questionnaire for Urinary Incontinence Diagnosis; BPMSES, The Broome Pelvic Muscle Self-Efficacy Scale; EQ5D-VAS, the EuroQol 5D-Visual Analogue Scale; Self-reported adherence (from 0 to 10, regarding their commitment to exercises where 0 means “no exercise at all” and 10 means “maximal adherence”)

### Risk of individual bias of the studies

**Fig. 2:**
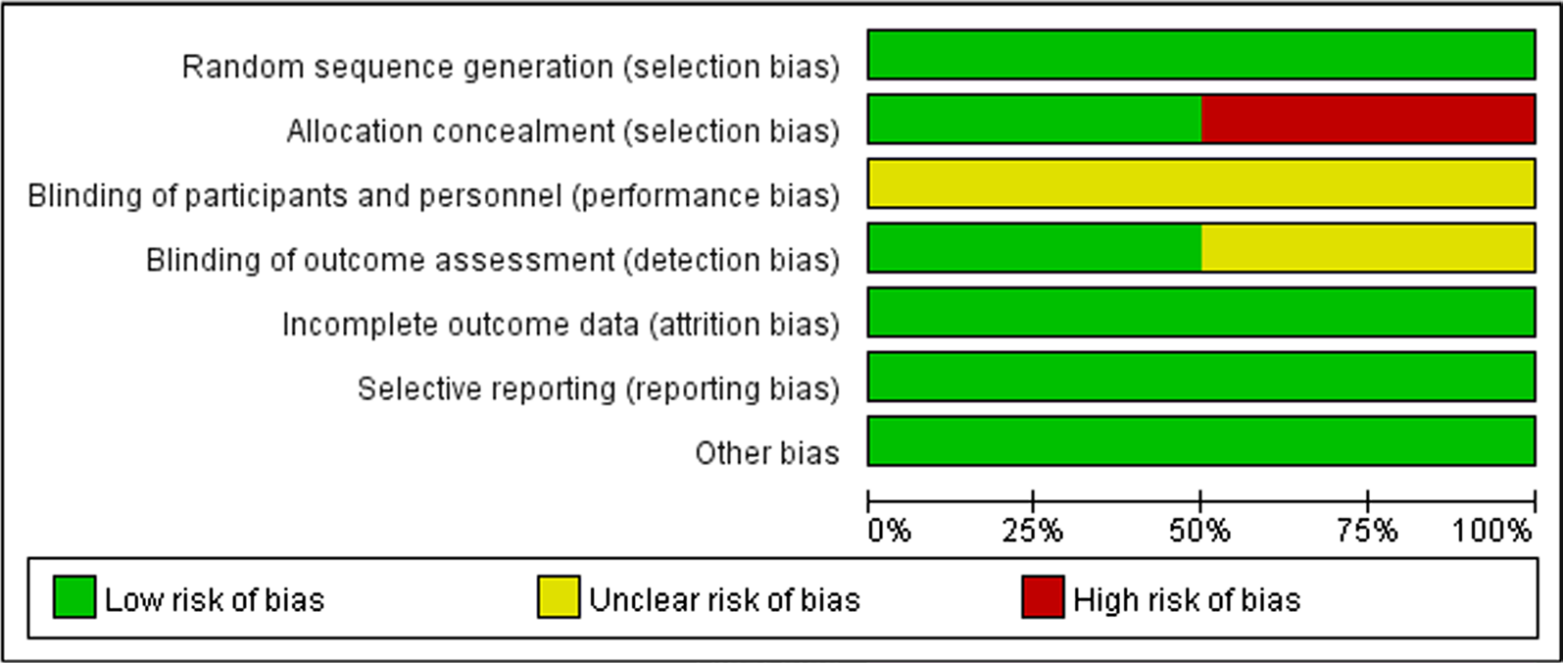
Risk of bias graph

**Fig. 3:**
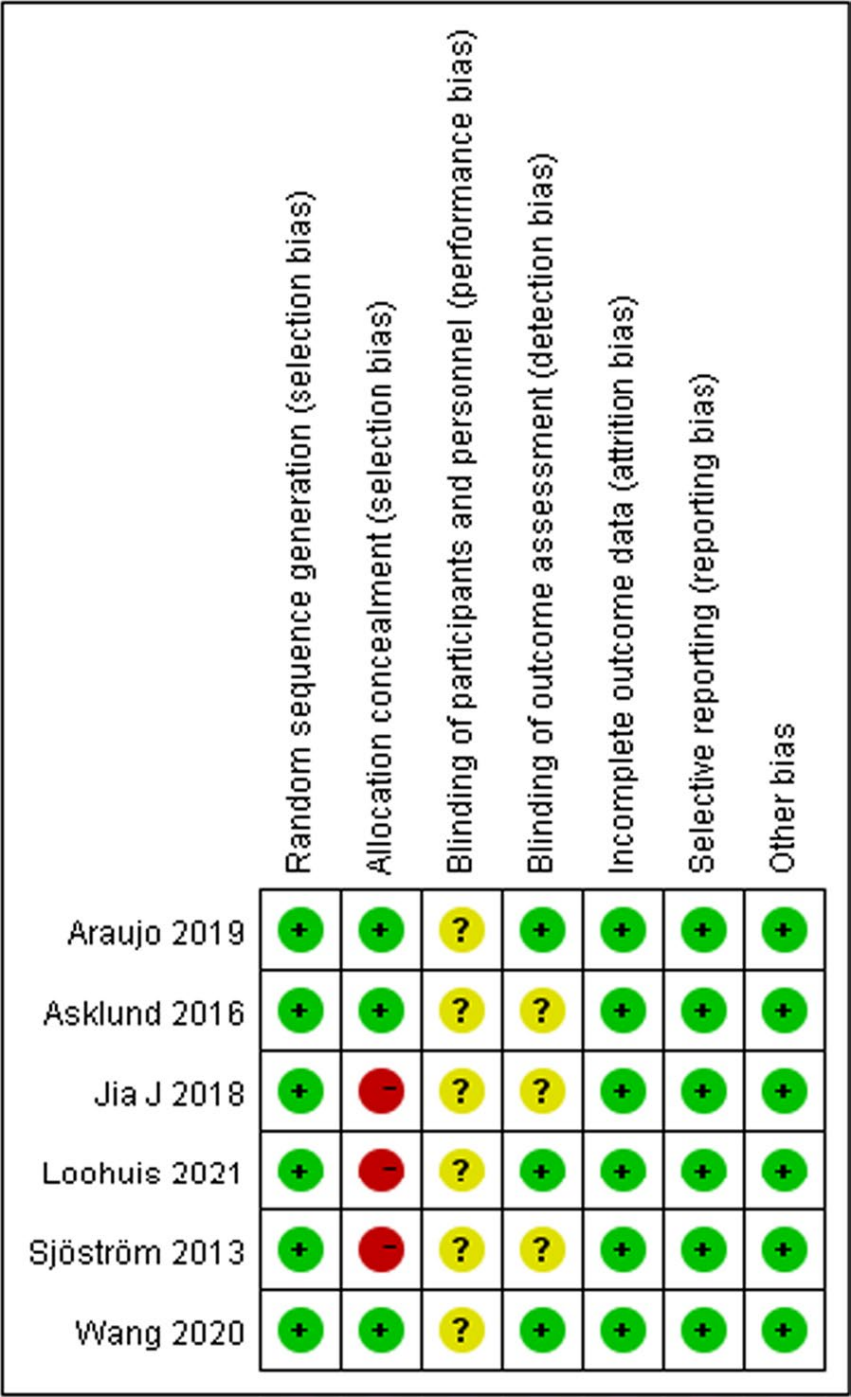
Risk of bias summary

The risk of bias graph and the risk of bias summary were shown in Figs. 2 and 3. For random sequence generation, six studies were assessed as low risk because the method of random sequence generation was described in detail in the original study [27–35]. Six studies reported adequate allocation concealment, three of which were assessed as low risk [29, 31, 33] and the other three as high risk [27, 32, 34]. For blinding of participants and intervention implementers, six studies were considered “not applicable” because intervention group participants cannot be blinded for mHealth app use. For the blinding of outcome evaluator, three studies were assessed as low risk [31, 33, 34], and the other three studies were considered unclear risk due to insufficient descriptions [27, 29, 32]. With regard to the assessment of incomplete outcome data, six studies were rated as low risk. Six studies were judged to be at low risk for reporting bias, and a low risk of other bias was given to six studies.

### Primary results of the eligible studies

This systematic review included six original studies, we extracted the data about the severity of symptoms, QoL and adherence, as showed in Tables 2, 3 and 4. Table 2 for example, at the same time point of follow-up, data from up to three studies used the same assessment tool. There were few data at the same time point, so this systematic review provided a narrative synthesis about symptoms severity, QoL and adherence.

**Table 2:** Summary of the results for assessment of the severity of symptoms based on ICIQ‐UI SF, ICIQ‐VS, and QUID

Studies	Assessment method	Assessment period																	
		Initial		1 month		6 weeks		2 months		3 months		4 months		6 months		1 year		2 years	
		Control	App	Control	App	Control	App	Control	App	Control	App	Control	App	Control	App	Control	App	Control	App
Loohuis et al. [34, 35]	ICIQ-UI-SF	10.3 ± 3.4	9.5 ± 3.2									− 2.6 ± 3.5^a^	− 2.2 ± 2.6^a^			7.1 ± 4.3	7.0 ± 3.3		
Wang et al. [33]	ICIQ-UI SF	6.1 ± 1.5	5.7 ± 1.4			2.0 ± 3.1	1.3 ± 2.9			1.0 ± 2.2	0.7 ± 2.0			0.4 ± 1.4	0.3 ± 1.1				
Araujo et al. [31]	ICIQ‐UI SF	15.9 ± 4.7	16.3 ± 4.0	12.4 ± 6.7	12.9 ± 4.6			11.3 ± 5.0	10.9 ± 6.9	9.7 ± 6.6	9.1 ± 6.6								
	ICIQ‐VS	13.7 ± 8.4	11.8 ± 8.8	10.9 ± 8.1	9.7 ± 8.5			7.0 ± 3.9	6.2 ± 7.9	6.0 ± 4.9	6.8 ± 8.2								
	QUID	15.6 ± 7.4	14.4 ± 8.3	9.2 ± 6.9	10.4 ± 9.4			4.5 ± 7.1	8.7 ± 9.3	3.9 ± 3.6	7.5 ± 9.0								
Jia et al. [32]	ICIQ‐UI SF	8.5 ± 2.3	8.5 ± 2.2											8.0 ± 2.0	4.4 ± 2.0				
Asklund et al. [29]	ICIQ-UI SF	11.0 ± 2.6	11.1 ± 3.0							10.2 ± 3.2	7.0 ± 3.5							None	8.1 ± 3.9
Sjöström et al. [27]	ICIQ-UI SF	10.3 ± 3.5	10.4 ± 3.1									7.3 ± 3.9	6.9 ± 3.1			6.7 ± 3.2	6.6 ± 3.1	6.4 ± 3.5	6.5 ± 3.0

^a^Change in outcomes from baseline to follow-Up in ICIQ‐UI SF score

None means the score was not mentioned in the original study

**Table 3:** Summary of the results for assessment of the condition-specific and health-specific QoL based on the ICIQ-LUTS QoL, ICIQ-VS QoL and EQ5D-VAS

Studies	Assessment method	Assessment period													
		Initial		1 month		2 months		3 months		4 months		1 year		2 years	
		Control	App	Control	App	Control	App	Control	App	Control	App	Control	App	Control	App
Loohuis et al. [34, 35]	ICIQ-LUTS QoL	33.4 ± 7.8	33.9 ± 8.3							− 3.8 ± 5.9^a^	− 4.3 ± 5.4^a^	29.1 ± 8.0	28.4 ± 6.0		
Araujo et al. [31]	ICIQ-VS QoL	5.9 ± 4.1	5.0 ± 4.6	3.9 ± 4.2	4.4 ± 4.3	3.1 ± 3.7	1.8 ± 3.2	1.3 ± 2.9	5.6 ± 4.3						
Asklund et al. [29]	ICIQ-LUTS QoL	34.8 ± 6.1	34.1 ± 6.1					34.1 ± 6.7	28.8 ± 6.4					None	30.2 ± 7.8
Sjöström et al. [27]	ICIQ-LUTS QoL	33.6 ± 8.2	33.6 ± 6.8							28.8 ± 7.3	27.8 ± 6.0	27.8 ± 5.7	27.5 ± 6.1	27.2 ± 6.4	26.5 ± 5.2
	EQ5D-VAS	79.2 ± 14.0	79.1 ± 13.6							81.8 ± 13.9	83.3 ± 10.3	83.4 ± 12.3	80.4 ± 13.7	83.5 ± 12.2	83.3 ± 13.2

None means the score was not mentioned in the original study

^a^Change in outcomes from baseline to follow-up in ICIQ-LUTS-QoL score

**Table 4:** Summary of the results for assessment of the adherence based on the BPMSES and self-reported adherence

Studies	Assessment method	Assessment period											
		Initial		1 month		6 weeks		2 months		3 months		6 months	
		Control	App	Control	App	Control	App	Control	App	Control	App	Control	App
Wang et al. [33]	BPMSES	None	None			55.5	59.9			None	62	51.5	60.4
Araujo et al. [31]	Self-reported adherence	None	None	8.3 ± 1.5	9.5 ± 0.7			9 ± 1.3	9.9 ± 0.2	8.7 ± 1.3	9.9 ± 0.2		
Jia et al. [32]	BPMSES	56.5 ± 22.1	53.8 ± 19.7									72.9 ± 17.5	105.4 ± 12.7

The average efficacy score was presented as mean ± SD or mean. None means the score was not mentioned in the original study

Table 2 provides the overall results of symptoms severity assessments based on the ICIQ-UI SF, ICIQ-VS and QUID. Loohuis et al. reported that the change in ICIQ-UI SF symptom score with mHealth app-based treatment (− 2.16 points) was noninferior to that with usual care (− 2.56 points), with a mean difference of 0.06 points between groups after 4 months of follow-up [34], and the ICIQ-UI SF score reduced from 9.5 to 7.0 in the mHealth app group and from 10.3 to 7.1 in the control group after 12 months of follow-up [35]. Wang et al. [33] reported that the ICIQ-UI SF score reduced from 5.7 to 0.3 in the mHealth app group and from 6.1 to 0.4 in the control group after 6 months of follow-up. Araujo et al. [31] showed that after 3 months of follow-up, the ICIQ-UI SF score decreased from 16.3 to 9.1 in the mHealth app group and from 15.9 to 9.7 in the control group, and the ICIQ-VS score reduced from 11.8 to 6.8 in the mHealth app group and from 13.7 to 6.0 in the control group, and finally the QUID score reduced from 14.4 to 7.5 in the mHealth app group and from 15.6 to 3.9 in the control group. Jia et al. [32] verified a reduction of the ICIQ-UI SF score from 8.5 to 4.4 in the mHealth app group and from 8.5 to 8.0 in the control group after 6 months of follow-up. Asklund et al. [29] verified that after 3 months of follow-up, the ICIQ-UI SF score decreased from 11.1 to 7.0 in the mHealth app group and from 11.0 to 10.2 in the control group, and Hoffman et al. [30] found that the ICIQ-UI SF score decreased from 11.1 to 8.1 in the mHealth app group after 2 years of follow-up [30]. Sjöstrom et al. showed that after 4 months of follow-up, the ICIQ-UI SF score decreased from 10.4 to 6.9 in the mHealth app group and from 10.3 to 7.3 in the control group [27], and after 2 years of follow-up, the ICIQ-UI SF score decreased from 10.4 to 6.5 in the mHealth app group and from 10.3 to 6.4 in the control group [28].

Table 3 shows summary results of the assessment of condition-specific and health-specific QoL to the SUI based on the ICIQ-LUTS QoL, ICIQ-VS QoL and EQ5D-VAS. Loohuis et al. reported that after 4 months of follow-up, the change in ICIQ-LUTS QoL score with mHealth app group (− 4.3 points) and usual care group (− 3.8 points), with a mean difference of − 0.57 points between groups [35], and the ICIQ-LUTS QoL score reduced from 33.9 to 28.4 in the mHealth app group and from 33.4 to 29.1 in the control group after 12 months of follow-up [36]. Asklund et al. [30] verified that after 3 months of follow-up, the ICIQ-LUTS QoL score reduced from 34.1 to 28.8 in the mHealth app group and from 34.8 to 34.1 in the control group, and Hoffman et al. [31] found that the ICIQ-LUTS QoL score decreased from 34.1 to 30.2 in the mHealth app group after 2 years of follow-up. Sjöstrom et al. showed that after 4 months of follow-up, the ICIQ-LUTS QoL score reduced from 33.6 to 27.8 in the mHealth app group and from 33.6 to 28.8 in the control group [28], and the ICIQ-LUTS QoL score reduced from 33.6 to 26.5 in the mHealth app group and from 33.6 to 27.2 in the control group after 2 years of follow-up [29]. Health-specific QoL was evaluated with the EQ5D-VAS [37]. After 4 months of follow-up, the EQ5D-VAS score improved from 79.1 to 83.3 in the mHealth app group and improved from 79.2 to 81.8 in the control group, and after 2 years of follow-up, the EQ5D-VAS score increased from 79.1 to 83.3 in the mHealth app group and from 79.2 to 83.5 in the control group. Araujo et al. [32] revealed that the ICIQ-VS QoL score increased from 5.0 to 5.6 in the mHealth app group and reduced from 5.9 to 1.3 in the control group after 3 months of follow-up.

We provided a detailed overview of the patient global impression of improvement of incontinence. Loohuis et al. reported that the majority of women in both the mHealth app-based treatment group (65.7%) and the usual care group (66.6%) had improved overall impressions after a follow-up of 4 months [34]. Asklund et al. [29] provided that the follow-up showed that mHealth app group participants reported much improved or very much improved urinary incontinence more often than control group participants, with an outcome of 91.8% in the mHealth app group. In the analysis of Sjöström et al. [27], participants in the mHealth app group rated their leakage as much better or very much better after treatment (40.9%), compared with participants in the control group (26.5%) after 4 months of follow-up. After a two-year follow-up [28], more participants in the mHealth app group believed that their leakage improvement was very high (39.2%) compared to the control group participants (23.8%).

### Secondary results of the eligible studies

Table 4 shows summary results for assessment of the adherence. Wang et al. [33] reported that the average efficacy score improved from 59.9 at 6 weeks to 62.0 at 3 months then declined slightly to 60.4 at 6 months in the mHealth app group, while in the control group, the average score declined continuously from 55.5 at 6 weeks to 51.5 at 6 months. Araujo et al. [31] reported that self-reported adherence rate (attribute a score, from 0 to 10) showed better results during the treatment, which increased from 9.5 to 9.9 in the mHealth app group after 3 months of follow-up. The study of Jia et al. [32] verified an increase in the BPMSES score from 53.8 (average) to 105.4 in the mHealth app group and from 56.5 to 72.9 in the control group after a follow-up of 6 months.

### Syntheses of results

Table 5 presented the SMD in ICIQ-UI SF, ICIQ-LUTS QoL and BPMSES scores for the control and mHealth app group in each eligible study. In the studies of Loohuis et al., Wang et al., Araujo et al., Asklund et al. and Sjöström et al. (both for the control group and the mHealth app group), the values of SMD/month for ICIQ-UI SF and ICIQ-LUTS QoL decreased as the follow-up time increased [27–29, 31, 33, 35]. Jia et al. showed that the SMD of BPMSES in the control and mHealth app group were 0.82 and 3.11, respectively, after 6 months follow-up [32].

**Table 5:** SMD in the scores of ICIQ-UI SF, ICIQ-LUTS QoL and BPMSES comparing initial value with the scores in post-intervention period

Studies	ICIQ-UI SF			ICIQ-LUTS QoL			BPMSES		
	Assessment period	SMD	SMD/month	Assessment period	SMD	SMD/month	Assessment period	SMD	SMD/month
*Loohuis et al. *[34, 35]									
Control	1 year	− 0.83	− 0.07	1 year	− 0.54	− 0.05			
App	1 year	− 0.78	− 0.07	1 year	− 0.72	− 0.06			
*Wang et al. *[33]									
Control	3 months	− 2.71	− 0.90						
App	3 months	− 2.90	− 0.97						
Control	6 months	− 3.93	− 0.66						
App	6 months	− 4.29	− 0.72						
*Araujo et al. *[31]									
Control	1 month	− 0.60	− 0.60						
App	1 month	− 0.79	− 0.79						
Control	2 months	− 0.95	− 0.48						
App	2 months	− 0.96	− 0.48						
Control	3 months	− 1.08	− 0.36						
App	3 months	− 1.32	− 0.44						
*Jia et al. *[32]									
Control	6 months	− 0.23	− 0.04				6 months	0.82	0.14
App	6 months	− 1.95	− 0.33				6 months	3.11	0.52
*Asklund et al. *[29]									
Control	3 months	− 0.27	− 0.09	3 months	− 0.11	− 0.04			
App	3 months	− 1.26	− 0.42	3 months	− 0.85	− 0.28			
Control	2 years	None	None	2 years	None	None			
App	2 years	− 0.86	− 0.04	2 years	− 0.56	− 0.02			
*Sjöström et al. *SPS:refid::bib27[27]									
Control	4 months	− 0.81	− 0.20	4 months	− 0.62	− 0.16			
App	4 months	− 1.13	− 0.28	4 months	− 0.90	− 0.23			
Control	1 year	− 1.07	− 0.09	1 year	− 0.82	− 0.07			
App	1 year	− 1.23	− 0.10	1 year	− 0.94	− 0.08			
Control	2 years	− 1.11	− 0.05	2 years	− 0.87	− 0.04			
App	2 years	− 1.28	− 0.05	2 years	− 1.17	− 0.05			

None means the score was not mentioned in the original study

*SMD* standardized mean difference

## Discussion

This systematic review aimed to summarize the evidence on the effectiveness of mHealth app-based PFMT on outcomes, including SUI symptom severity, QoL, and the patient's global impression of improvement. In addition, this systematic review summarized the evidence on the impact of mHealth app-based PFMT on adherence. We found that mHealth app-based PFMT showed positive effects on primary and secondary indicators. Compared with control group, the mHealth app group had significant improvement in severity of SUI symptoms, QoL and the global impression of patients. The mHealth app group also showed a significant improvement in adherence to PFMT.

PFMT is a first-line strategy for SUI [36]. One possible way to meet the future needs of the medical industry is to enhance patients' self-management capabilities through mHealth app [37, 38]. A number of studies show that mHealth app improve health outcomes [39]. MHealth app are potentially effective for delivering PFMT to women, which is convenient, flexible, and time-saving [40]. Use of mHealth app for PFMT expands access to care and aids the management of patients [27].

### Effects of mHealth app-based PFMT on primary objectives

#### The severity of SUI symptoms

In this systematic review, all original studies that met the inclusion criteria used ICIQ-UI SF to analyze the severity of SUI symptoms. Based on this, when comparing participants in the mHealth app group with those in the control group, a significant decrease in ICIQ-UI SF scores was seen. The summary results of all RCTs and quasi-randomized controlled trials showed that mHealth app-based PFMT improved the severity of SUI symptoms, which is consistent with other studies [18, 41].

#### QoL

SUI can affect the QoL. This systematic review showed that mHealth app-based PFMT exercises are effective in treating SUI and will improve the QoL of patients. This was probably due to the fact that these women have less SUI symptoms than before and enhanced their self-confidence in daily activities [42].

#### The patient’s global impression of improvement

The PGI-I is an outcome measure that is readily understood by both the patient and the clinician, and it gives a direct reflection of the patient's overall opinion. The follow-up PGI-I results in our systematic review showed that mHealth app group participants reported much improved. This could be associated with improved urinary symptoms and QoL.

### Effects of mHealth app-based PFMT on secondary objective

#### Adherence

MHealth app-based PFMT works better among people who are interested in it and have higher expectations [41], it has the potential to improve adherence of PFMT [42]. The eligible studies for adherence assessment used the BPMSES and self-reported adherence rate, verifying an increase in the self-efficacy scores of patients in the mHealth app group [32, 33]. This could be explained by the mHealth app group exhibited greater self-efficacy across the follow-up, indicating the efficacy of the mHealth app in improving and maintaining adherence to training. Evidence suggests that participants with higher self-efficacy are more likely to seek out and stick to PFMT protocols [42].

This systematic review assessed the SMD of the ICIQ-UI SF, ICIQ-LUTS QoL and BPMSES scores, comparing initial values with scores in the post-intervention period. In most studies, we found that the SMD was higher in mHealth app group for the ICIQ-UI SF, ICIQ-LUTS QoL and BPMSES than in the control group [27–29, 31–33]. But the values of SMD/month reduced as the follow-up time increased, it may be associated with the sample loss over time, which is consistent with previous study [18].

### Strengths and limitations

Strengths of this systematic review are as follows. First, it offers a literature review on the impact of mHealth app-based PFMT on SUI or stress-predominant MUI. Currently, only two systematic reviews have evaluated the impact of mHealth app-based PFMT on urinary incontinence, but these were limited to qualitative analyses of the results and included an insufficient number of articles. Our systematic review included more studies to evaluate multiple outcomes of the impact of mHealth app-based PFMT on SUI or stress-predominant MUI, including the severity of SUI symptoms, QoL, the patient's global impression of improvement and adherence. Second, we searched a total of 12 databases and systematically reviewed articles using an integrated search strategy, ultimately including six studies.

However, this study also has some limitations. Six studies that meet the inclusion criteria, and three of these trials conducted follow-up studies of the same original population for several years [27–30, 34, 35], we completed a narrative analysis. Future research should aim to capture more high quality RCTs to allow for statistical analysis of effects on outcomes.

## Conclusion

This systematic review determined that mHealth app-based PFMT showed promise from the perspective of improving both outcomes and exercise adherence.

## Data Availability

All data generated or analyzed during this study are included in this published article.

## Abbreviations

PFMT: Pelvic Floor Muscle Training
SUI: Stress Urinary Incontinence
MUI: Mixed Urinary Incontinence
QoL: Quality of Life
RCTs: Randomized Controlled Trials
ICIQ‐UI SF: International Consultation on Incontinence Questionnaire-Urinary Incontinence Short Form
ICIQ‐VS: International Consultation on Incontinence Questionnaire-Vaginal Symptoms
QUID: Questionnaire for Urinary Incontinence Diagnosis
ICIQ-LUTS QoL: International Consultation on Incontinence Questionnaire-Lower Urinary Tract Symptoms Quality of Life
EQ5D-VAS: The EuroQol 5D-Visual Analogue Scale
BPMSES: The Broome Pelvic Muscle Self-Efficacy Scale
PGI-I: Patient's Global Impression of Improvement
SMD: Standardized Mean Difference
mHealth app: Mobile Health Applications
